# Analysis of stakeholders networks of infant and young child nutrition programmes in Sri Lanka, India, Nepal, Bangladesh and Pakistan

**DOI:** 10.1186/s12889-017-4337-1

**Published:** 2017-06-13

**Authors:** Shahadat Uddin, Hana Mahmood, Upul Senarath, Quazi Zahiruddin, Sumit Karn, Sabrina Rasheed, Michael Dibley

**Affiliations:** 1 Complex Systems Research Group, Faculty of Engineering & IT, The University of Sydney, Darlington, Australia; 2 Maternal, Neonatal and Child Health Research Network, International Research Force, Islamabad, Pakistan; 3 Faculty of Medicine, University of Colombo, Colombo, Sri Lanka; 4 Centre of Excellence School of Epidemiology and Public Health, Datta Meghe Institute of Medical Sciences, Nagpur, India; 5 Food and Agriculture Organization of the United Nations, Kathmandu, Nepal; 6 International Centre for Diarrhoeal Disease Research, Dhaka, Bangladesh; 7 Menzies Centre for Health Policy, Sydney School of Public Health, The University of Sydney, Darlington, Australia

## Abstract

**Background:**

Effective public policies are needed to support appropriate infant and young child feeding (IYCF) to ensure adequate child growth and development, especially in low and middle income countries. The aim of this study was to: (i) capture stakeholder networks in relation to funding and technical support for IYCF policy across five countries in South Asia (i.e. Sri Lanka, India, Nepal, Bangladesh and Pakistan); and (ii) understand how stakeholder networks differed between countries, and identify common actors and their patterns in network engagement across the region.

**Methods:**

The Net-Map method, which is an interview-based mapping technique to visualise and capture connections among different stakeholders that collaborate towards achieving a focused goal, has been used to map funding and technical support networks in all study sites. Our study was conducted at the national level in Bangladesh, India, Nepal, and Sri Lanka, as well as in selected states or provinces in India and Pakistan during 2013–2014. We analysed the network data using a social network analysis software (NodeXL).

**Results:**

The number of stakeholders identified as providing technical support was higher than the number of stakeholders providing funding support, across all study sites. India (New Delhi site – national level) site had the highest number of influential stakeholders for both funding (43) and technical support (86) activities. Among all nine study sites, India (New Delhi – national level) and Sri Lanka had the highest number of participating government stakeholders (22) in their respective funding networks. Sri Lanka also had the highest number of participating government stakeholders for technical support (34) among all the study sites. Government stakeholders are more engaged in technical support activities compared with their involvement in funding activities. The United Nations Children’s Emergency Fund (UNICEF) and the World Health Organization (WHO) were highly engaged stakeholders for both funding and technical support activities across all study sites.

**Conclusion:**

International stakeholders were highly involved in both the funding and technical support activities related to IYCF practices across these nine study sites. Government stakeholders received more support for funding and technical support activities from other stakeholders compared with the support that they offered. Stakeholders were, in general, more engaged for technical support activities compared with the funding activities.

## Background

Lack of appropriate infant and young child feeding (IYCF) practices is a significant contributor to child mortality rates worldwide. According to the World Health Organization (WHO), undernutrition is associated with 45% of child deaths [1]. These adverse effects of poor IYCF practices and undernutrition are worst for the poorest populations. In order to reduce the adverse effects of inappropriate IYCF and child undernutrition in this region, different non-government and academic organisations have been engaged in five South Asian countries (i.e. Sri Lanka, India, Nepal, Bangladesh and Pakistan) under the banner of the South Asian Infant Feeding Research Network (SAIFRN). As a part of their research mandate, SAIFRN used a social network research approach to understand the level of participation and engagement of different international and domestic stakeholders in relation to funding and technical support activities for infant and young child nutrition (IYCN) in these five South Asian countries.

**Fig. 1:**
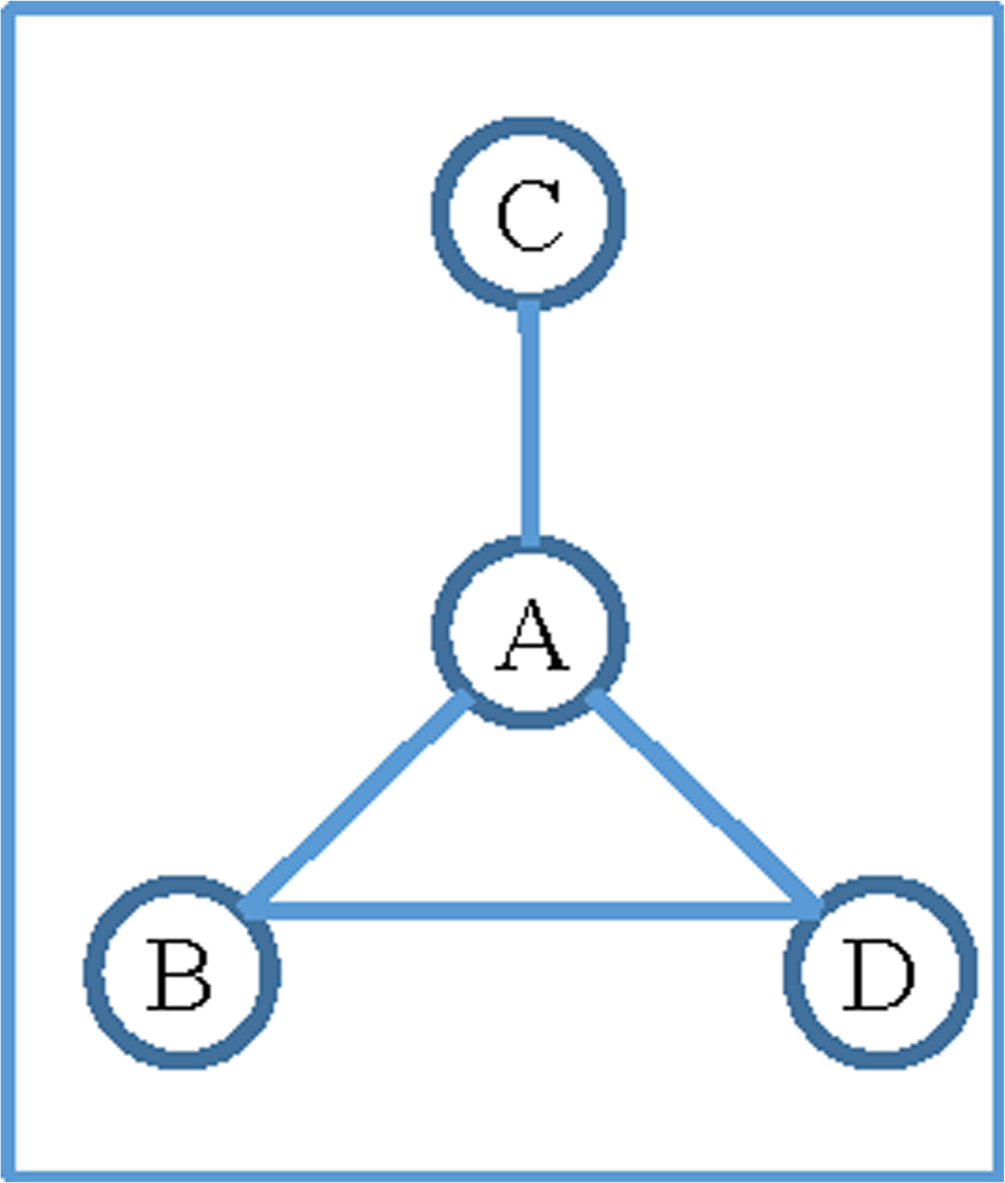
A friendship network among four individuals

A social network is a group of actors that are linked together by a set of social relations [2]. These relations describe the ties of a specific kind among the actors of the network. Actors of a social network can be individuals, organisations or companies. Regardless of what they are, they are always the smallest single unit inside a network. In a visual illustration of a social network, actors are presented by nodes and relations among actors are presented by links. For example, Fig. 1 shows a friendship network where actors are the four individuals who are represented by four nodes labelled with *A*, *B*, *C* and *D*. The links among them represent their friendship ties. Social networks can also be thought of as neighbourhoods since networks are comprised of the actors and the relationships between those actors. The formation of a social network is typically associated with the need for an actor to receive some sort of information or resource from others; thus creating an exchange whereby actors invest in relationships determined by their level of needs [3].

The key principles that make the social network as a distinct research perspective within the social and behavioural sciences are: (i) actors and their actions in a social network are viewed as interdependent rather than independent, autonomous units; and (ii) relational ties between actors are channels for transfer of resources (either material or non-material) [2]. In order to explore a health related social science research question (e.g. women’s health care seeking behaviour in pregnancy in a low socioeconomic urban community), a standard social science research approach usually defines a population of relevant units (e.g. pregnant women), takes a random sample of them, if the population is quite large, and then measures a variety of characteristics (e.g. education, household economic status, previous behaviours, and residence). The key assumption for such a social science approach is that the behaviour of a specific unit does not influence any other units; and thus, ignores the relational information among underlying units of the context [4]. Unlike social science approaches, a social network approach always gives importance to the relationships among units in a study. For this reason, a social network does not emphasise individual actors and their attributes, instead it focuses on the relations among actors. The principal task in a social network study is therefore to understand structural properties (e.g. which individual is highly connected within the network) of social units and how these structural properties influence observed characteristics (e.g. decision-making skill) and associations among characteristics.

The location of an actor inside a social network can be an indicator of the strength of ties associated with that actor. An individual near the centre of a friendship network often has more ties or links between herself and the other actors, as opposed to someone on the outer fringes of that friendship network. A person on the outer edge of the network could be connected to the network by only one link. A social network analysis is a commonly followed analytical process in the ‘*Network Science*’ literature, which is used to map the connections among actors and measure and visualise their relationships in a social network [5]. A social network analysis of a social network provides both a visual and a mathematical analysis of network relations among actors within that network. Because of its ability to assess connectivity patterns of networks and network behaviour of their member actors, the usefulness of the application of social network analysis has already been appreciated across many disciplines, including health analytics [6], disease prediction [7, 8], co-author network [9], organisational science [10, 11] and anthropology [3, 12].

In order to represent the description of networks compactly and systematically, the social network analysis approach follows both graphical and mathematical techniques. Graphical techniques are used to visualise a given social network in terms of nodes and their connections. On the other side, mathematical techniques are applied to explore the structural properties of social networks.

Using graphical techniques of social network analysis, many network and non-network characteristics of actors and edges can be visualised. Researchers mostly use label, size, shape and colour of actors and thickness of edges in order to visually represent various non-network and network characteristics of actors of a social network [2, 13]. Labels are usually used to indicate names of the underlying actors. The size of an actor in a social network can be used to represent its network characteristic (e.g. degree centrality). If the actor has a higher degree centrality then its size will be bigger and vice versa. The shape and colour of actors in a social network can be used to represent both non-network and network characteristics of actors. In order to visualise an inter-organisational network, for example, square and circle shapes may be used to represent all government and non-government organisations, respectively. For the same purpose, anyone can use two different colours instead of two different shapes. On the other side, any of these two visual features (i.e. shape and colour) can be used to represent groups of actors that share similar structural properties. A set of actors form a network community if they are densely connected among themselves and sparsely connected with other network actors. All member actors of a social network community can be presented, for example, by the same colour or shape. The thickness of an edge that connects two actors can be used, for example, to represent the strength of relations between those actors.

Based on diverse mathematical techniques, researchers proposed many social network analysis measures to quantify structural properties of the actors of a network and the network itself [2, 14]. These measures have successfully been applied to explore networks and their participants by evaluating locations of actors in networks (e.g. [13]). One of the basic measures of the social network analysis is the network centrality, which is a structural attribute of nodes in a network. This attribute determines the relative importance of an actor within a network (e.g. *how important a person is as an advice provider within her friendship network* or *how well-used a road is within an urban network*). The selection of social network measures to study a network mainly depends on the social network research question under consideration. There are three primary measures of the network centrality: (i) degree centrality (representing activity of actors and their popularity in a network); (ii) representing closeness centrality (reachability of actors from other actors in a network); and (iii) betweenness centrality (representing actors’ control over the information flow in a network). Each of these measures addresses different structural characteristics associated with actors to assess their level of centralisation within the network.

This article follows a social network analysis approach to explore funding and technical support networks of IYCN programmes in Sri Lanka, India, Nepal, Bangladesh and Pakistan. In particular, four measures of social network analysis (in-degree, out-degree, closeness and betweenness) have been used to analyse funding and technical support networks among different stakeholders in these five South Asian countries.

## Methods

During the course of this research, the Australian Agency for International Development (AusAID) was absorbed into the Department of Foreign Aids and Trade (DFAT). For this reason, this article considered AusAID/DFAT as a single organisation in reporting its results.

### Research data collection

In order to understand the role of IYCN actors in different stakeholder networks across five South Asian countries (Sri Lanka, India, Nepal, Bangladesh and Pakistan), a participatory tool know as Net-Map was used to capture networks among different types of stakeholders within these five countries in relation to funding and technical support, in 2013–2014. The Net-Map is an interview tool, which was developed by the International Food Policy Research Institute (IFPRI) [15]. It was used to interview representatives of different stakeholders to map their network connectivity related to funding and IYCN technical support activities. Local research teams in each country were trained in the Net-Map method by IFPRI staff, and collected data at the national level in Sri Lanka, Nepal and Bangladesh, and at both national level and in two selected states/provinces in each of India (Maharashtra and Andhra Pradesh) and Pakistan (Sindh and Punjab). The required ethics approvals were obtained before conducting the research from the relevant authorities of each country.

In each of the Net-Map interviews, the following two questions were asked in order to capture the funding and technical support networks among different stakeholders: (i) who plays a role in shaping policy and program decisions on IYCN at the national/provincial level in the country?; and (ii) who provides funding and technical assistance to whom as a means of engaging in or influencing policy and programme decisions on IYCN? Responses from the first question can identify the important actors that play an important role in the corresponding stakeholder networks. Responses from the second question can map the connections between any pair of actors either in the funding network and/or technical support network. Most of the interviews were audio recorded upon taking consent from interviewees and were conducted by a moderator with two to three support staff. The recorded activities were transcribed and translated into English. Although we collected both qualitative and quantitative data, this paper draws on only the quantitative data.

Data was collected in all the study sites in the form of group interviews. The major challenges in such a methodology is to make sense of massive amounts of data, reduce the volume to valuable information, identify significant patterns and constructing a framework to communicate the essence of what the data reveals. Certain biases arose throughout process. We managed to avoid consistency bias, error bias and reference bias by devising the interview guide neutrally with clear definitions of terminologies used to address the research question. Sampling bias was addressed by including respondents from all segments of stakeholders for IYCF including academia, researchers, development partners, government bodies and media. This was also addressed by conducting discussions in locations accessible to respondents. Although participation from all segments was not equal, the inclusion criterion of the respondents was such that they were expert, professional and highly knowledgeable with respect to the IYCN environment in their respective countries. Data was triangulated by cross referencing both the qualitative and quantitative data. Quantitative data was entered twice by two separate researchers into excel sheets.

### Network measures of social network analysis

As mentioned earlier, to capture the importance of network positions of different stakeholders in the corresponding funding and technical support networks, this study used four measures of social network analysis. They are in-degree centrality, out-degree centrality, closeness centrality and betweenness centrality, and are described below. These basic centrality measures have been used extensively in the healthcare literature to quantify the network positions of individual actors in networks (e.g. [13, 16, 17]).

**Fig. 2:**
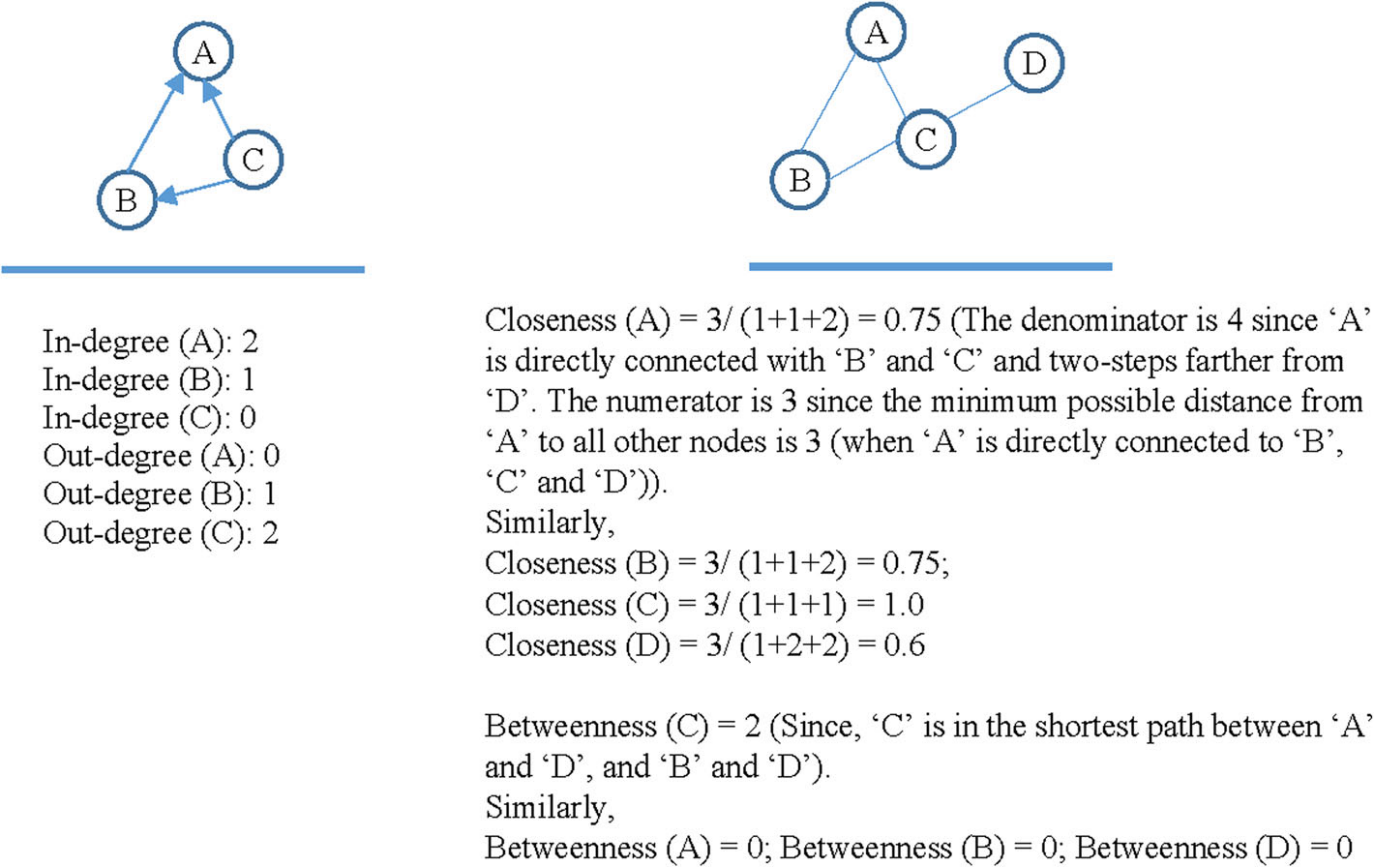
Illustration of the calculation of four different centrality measures using abstract network data

The first centrality measure (*degree centrality*) indicates the activity of actors and their popularity in a network. An actor with a high degree centrality in a social network has a high level of direct connections with other network actors [2]. It can be of two types: in-degree centrality and out-degree centrality. *In-degree centrality* quantifies the tendency of an actor to receive ‘choices’ from the other network actors [2]. Here, ‘choices’ indicate the intention of other actors to form a link with the actor under consideration. In other words, in-degree centrality is a measure of receptivity or popularity. *Out-degree centrality* quantifies the tendency of an actor to make ‘choices’ in terms of forming links with other network actors [2]. In other words, out-degree centrality is a measure of expansiveness or activity of an actor in a network. A high out-degree centrality for an organisation in a policy network indicates that it influences other stakeholders through technical support and funding support. Conversely, a high in-degree centrality for an organisation reveals that it has been influenced highly by other stakeholders. The second centrality measure (*closeness centrality*) represents the reachability of an actor from the other actors in a network [2]. An actor is said reachable from another actor if there is a path linking the two actors. The third centrality measure (*betweenness centrality*) represents the capacity of an organisation to control the flow of information in the network (for example, technical and funding support) between any pair of the other organisations in the policy network [2]. The underlying assumption of the betweenness centrality is that ‘actors in the middle’ have more ‘inter-organisational influence’ on the others in a network [18]. A further explanation regarding how to calculate each of these four network centrality measures can be found in Fig. 2.

## Results and discussion

### Difference in network size

**Fig. 3:**
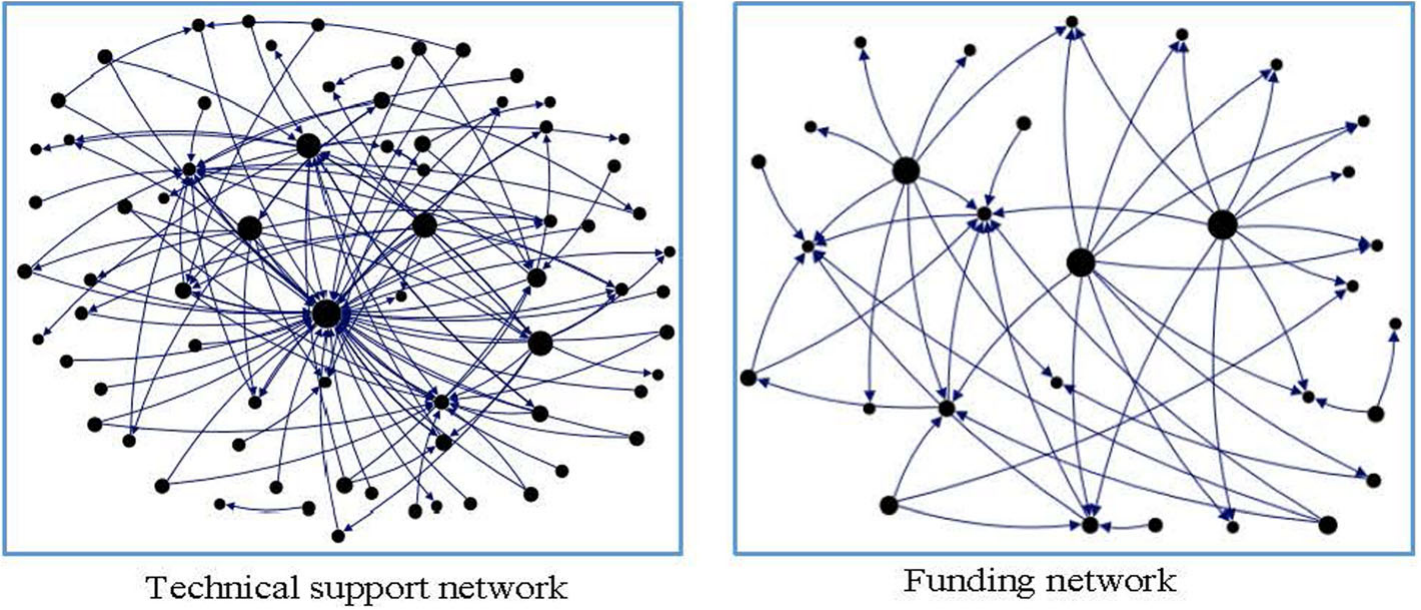
A visual illustration of the difference between technical support and funding network for Bangladesh. Nodes represent different identified stakeholders in this study site. The size of a node is proportional to its out-degree (i.e. activity of a node in a network) in both networks. For the other eight study sites, we observed similar visual differences between technical support and funding networks

As revealed in Table 1, for each study site the size of the technical support network (the total number of participating organisations in the network) was larger than that of the funding network, and these differences were statistically significant (*p* < 0.01). Figure 3 shows a visualisation of such differences for the technical and funding networks of Bangladesh. India at a national level had the largest network size for both its funding (43 organisations) and technical support (86 organisations) networks. Pakistan at the federal level had the smallest network size for both its funding (9 organisations) and technical support (15 organisations) networks. Stakeholders engaged more with other organisations for technical support activities compared to funding activities.

**Table 1:** Number and percentage of government stakeholders in the infant and young child nutrition (IYCN) funding and technical support networks as identified from Net-Map interviews at each country, state or provincial site

Study Site	Funding Network		Technical Support Network	
	Network Size	Number (%) of Government Stakeholder	Network Size	Number (%) of Government Stakeholder
India at National Level	43	22 (51%)	86	32 (37%)
India–Maharashtra	31	8 (26%)	62	22 (35%)
India–Andhra Pradesh	32	10 (31%)	43	15 (35%)
Pakistan at Federal Level	9	5 (56%)	15	7 (47%)
Pakistan–Sindh	11	4 (36%)	34	8 (24%)
Pakistan–Punjab	34	10 (29%)	44	17 (39%)
Sri Lanka	36	22 (61%)	57	34 (60%)
Nepal	28	8 (29%)	43	17 (40%)
Bangladesh	30	5 (17%)	72	18 (25%)

The number of actors in a network is the ‘network size’ for that network

### Participation of government stakeholders

Government stakeholders were engaged in both the funding and technical support networks across all study sites (see third and fifth columns of Table 1). The number of government stakeholders was higher in the technical support than in the funding networks at each study site, and these differences were statistically significant (*p* < 0.01). Among all the funding networks, India at a national level and Sri Lanka had the highest number of government stakeholders (each with 22 stakeholders), and Sindh (Pakistan) site had the lowest number of government stakeholder (4 stakeholders). Expressed as a percentage, Sri Lanka had the highest percentage (61%) while Bangladesh had the lowest (17%) percentage of government stakeholders in their respective funding networks. Among all the technical support networks, Sri Lanka had the highest number of government stakeholders (34 stakeholders) and Pakistan at the federal level had the lowest number of government stakeholders (7 stakeholders). Sri Lanka also had the highest percentage of government stakeholders (60%), while, Sindh (Pakistan) had the lowest percentage of government stakeholders in their technical support networks. Table 2 shows major government stakeholders that have been playing a significant role in IYCF activities in each of the nine study sites. As revealed in this table, there are few government stakeholders that have been playing major role in respect to all centrality measures for funding and/or technical support activities. For example, Ministry of Health and Family Welfare (MoHFW) has been found as one of the 10 most contributors in respect to all four centrality measures in both funding and technical support networks for the India (at National level) study site. Other government stakeholders that play a major role in respect to all centrality measures in funding and/or technical support activities are highlighted using italic font in Table 2. Government stakeholders are found more in the top-10 list of technical support activities compared with the funding activities (compare columns 6–9 against columns 2–5). Overall, top-10 government stakeholders received more funding (compare columns 2 and 3) and technical supports (compare columns 6 and 7) from other stakeholders compared with their offerings of these two types of supports to other stakeholders. India (Maharashtra) and Sri Lanka have the highest number of government stakeholders (5) in the top-10 list that offer funding support to other stakeholders (column 3). No government stakeholder from Nepal was found as one the 10 top stakeholders that offer funding support to other stakeholders (the empty cell in column 3). Sri Lanka also has the highest number of government stakeholders (6), followed by India–Maharashtra (5) and India–Andhra Pradesh (5), in the top-10 list of stakeholders that offer technical support to other stakeholders (column 7).

**Table 2:** List of government stakeholders that have been playing a significant role for infant and young child nutrition (IYCN) activities in each study site. This table includes only those government stakeholders that are found as one of the 10 top contributors to IYCN activities in respect to the corresponding network centrality measure. In each cell, stakeholders are ordered as per their chronological orders in the top-10 list. Common stakeholder(s) across cells of either funding or technical support category are highlighted using italic font

Study Site	Funding Network				Technical Support Network			
	*In-degree centrality*	*Out-degree centrality*	*Betweenness centrality*	*Closeness centrality*	*In-degree centrality*	*Out-degree centrality*	*Betweenness centrality*	*Closeness centrality*
India at National Level	*MoHFW*; *MoWCD*; NACO; and AIIMSP	*MoHFW*; *MoWCD*; ICMR; and MoF	*MoHFW*; *MoWCD*: ICMR; and MoF;	*MoHFW*; MoF; and *MoWCD*	*MoWCD*; *MoHFW*; *PC*; and PMO	*MoWCD*; *MoHFW*; and *PC*	*MoWCD*; *MoHFW*; *PC*; FSSAI; and PMO	*MoWCD*; *MoHFW*; *PC*; FSSAI; and PMO
India - Maharashtra	*MoHFW*; *MoWCD/ICDS*; PHD; and RJNM	MoF; PC; *MoHFW*; *MoWCD/ICDS*; and NRHM/RMNCH + A	*MoHFW*; PC; *MoWCD/ICDS*; and MoF	*MoHFW*; PC; *MoWCD/ICDS*; MoF; and NRHM/RMNCH + A	*MoHFW*; *MoWCD/ICDS*; PHD; NRHM/RMNCH + A; RJNM; MoSW; PC; and MoTD	*MoWCD/ICDS*; NB; FNB; *MoHFW*; and MoSW	*MoHFW*; *MoWCD/ICDS*; PHD; NB; PC; PMO; and NRHM/RMNCH + A	*MoHFW*; *MoWCD/ICDS*; PHD, MoSW; NB; PC; and PMO
India–Andhra Pradesh	*WDCW*; AP Foods; and MEPMA	*WDCW*; and NRHM	*WDCW*	MEPMA; NRHM; and *WDCW*	WDCW; *MoHFW*; AP Foods; *FNB*; MEPMA; and *NRHM*	*FNB*; *MoHFW*; AP Foods; and *NRHM*	WDCW; *MoHFW*; *FNB*; *NRHM*; and AP Foods	WDCW; *FNB*; *MoHFW*; and *NRHM*
Pakistan at Federal Level	NNW; LHWP; MNCHP; and MoH	P&D	NNW; LHWP; and MNCHP	P&D; MoH; NNW; LHWP; and MNCHP	*NNW*; LHWP; MNCHP; LAWMIN; MoPW; PARL; and MoH	*NNW*; and MoH	*NNW*; LHWP; and MNCHP	*NNW*; MoH; LAWMIN; LHWP; and MNCHP
Pakistan –Sindh	PN&D; N CELL; HRSU; and *HDEPTT*	*HDEPTT*	PN&D; N CELL; *HDEPTT*; and HRSU	PN&D; N CELL; *HDEPTT*; and HRSU	*HDEPTT*; *HRSU*; and N CELL;	*HRSU*; and *HDEPTT*	*HDEPTT*; and *HRSU*	*HDEPTT*; and *HRSU*
Pakistan–Punjab	HDEPTT; LHWP; ADEPTT; and FDEPTT	HDEPTT	HDEPTT	ADEPTT; and FDEPTT;	HDEPTT; IP; ADEPTT; PHED; and FDEPTT	PCPD	HDEPTT; IP; PFA; and ADEPTT	HDEPTT; IP; and PHED
Sri Lanka	*FHB*; MRI; NSACP; MoIM; and MoFS	MoH; *FHB*; MoF; SH; and DDG PHS 2	MoH; *FHB*; DoI; MoF; DGHS; MRI; and MoIM	MoH; *FHB*; MRI; NSACP	*FHB*; MoIM; DDG PHS 1; CMC; NNC; and *MRI*	*FHB*; *MRI*; MoH; HEB; SH; and NCD	*FHB*; MoH; *MRI*; MoIM; HEB; DDG PHS 1; SH; and CMC	*FHB*; *MRI*; MoH; MoIM; HEB; and DDG PHS 1
Nepal	NuTEC/CHD; DFTQC; and NHEICC		NuTEC/CHD; and DFTQC	NuTEC/CHD	*NuTEC/CHD*; DFTQC; and NPC	*NuTEC/CHD*	*NuTEC/CHD*; and DFTQC	*NuTEC/CHD*; and DFTQC
Bangladesh	*IPHN/NNS*	MoHFW; and *IPHN/NNS*	*IPHN/NNS*	*IPHN/NNS*	*IPHN/NNS*; *MoHFW*; and *DGHS*	*IPHN/NNS*; *DGHS*; and *MoHFW*	*IPHN/NNS*; *MoHFW*; and *DGHS*	*IPHN/NNS*; *MoHFW*; and *DGHS*

*ADEPTT* Agriculture Department, *AIIMSP* All Indian Institute of Medical Sciences, Patna, *AP Foods* Andhra Pradesh Foods, *CMC* Code Monitoring Committee, *DDG PHS* Deputy Director General of Public Health Services, *DFTQC* Department of Food, Technology and Quality Control, *DGHS* Director General of Health Services, *DoI* Department of Information, *FDEPTT* Food Department, *FHB* Family Health Bureau, *FNB* Food and Nutrition Board, *FSSAI* Food Safety and Standards Authority of India, *HDEPTT* Department of Health, *HEB* Health Education Bureau, *HRSU* Health System Reforms Unit, *ICMR* Indian Council of Medical Research, *ICDS* Integrated Child Development Services, *IP* Integrated Program, *IPHN/NNS* Institute of Public Health Nutrition/National Nutrition Services, *LAWMIN* Law Ministry, *LHWP* Lady Health Workers Program, *MEPMA* Mission for Elimination of Poverty in Municipal Areas, *MNCHP* Maternal, Neonatal and Child Health Program, *MoF* Ministry of Finance, *MoFS* Ministry of Fisheries, *MoH* Ministry of Health, *MoHFW* Ministry of Health and Family Welfare, *MoIM* Ministry of Indigenous Medicine, *MoPW* Ministry of Population Welfare, *MoSW* Ministry of Social Welfare, *MoTD* Ministry of Tribal Development, *MoWCD* Ministry of Women and Child Development, *MRI* Medical Research Institute, *N CELL* Nutrition Cell, *NACO* National Aids Control Organisation, *NB* Nutrition Bureau, *NCD* Nutrition Coordination Division, *NHEICC* National Health, Education, Information and Communication Center, *NNC* National Nutrition Council, *NNW* National Nutrition Wing, *NRHM* National Rural Health Mission, *NSACP* National STD/AIDS Control Program, *NuTEC/CHD* Nutrition Technical Committee/Child Health Division, *P&D* Planning & Development, *PARL* Parliament, *PC* Planning Commission, *PCPD* Punjab Consumer Projection Department, *PFA* Punjab Food Authority, *PHD* Public Health Department, *PHED* Public Health Engineering Department, *PMO* Prime Minister’s Office, *PN&D* Planning & Development Department, *RJNM* Rajmata Jijau Nutrition Mission, *RMNCH + A* Reproductive, Maternal, Newborn, Child and Adolescent Health, *SH* Secretary Health, *WDCW* Women Development & Child Development

No significant difference was noticed in the engagements of government stakeholders in different study locations that have different structure of decentralisation. Both Indian and Pakistan have different government structure (i.e. State/Province) compared with the other three countries. There are more government stakeholders in the top-10 lists of both funding and technical support activities in the Maharashtra site of India compared with its Federal and Andhra Pradesh study sites (Table 2) but this difference is not statistically significant. For other six study sites, this study did not notice any such pattern of engagement for government stakeholders.

### Engagement of international stakeholders

Table 3 provides a summary statistics of the engagement of 14 major international stakeholders in relation to the funding and technical support activities across all study sites. In this table, an organisation has been marked with “blank” for a particular funding network or technical support network only if that organisation has not been identified as an important contributor from the Net-Map interviews. An “X” mark indicates that the organisation has been found as an important contributor in relation to the corresponding network measure in the funding network, or the technical support network, but not as one of the 10 top contributors. If an organisation has been found as one of the 10 top contributors for a network measure then that organisation has been marked with its position among the 10 top contributors against that network measure.

**Table 3:** Engagement of major international stakeholders in infant and young child nutrition (IYCN) activities with other organisations across different study sites: (a) in Funding network; and (b) in Technical support network

	India												Pakistan												Sri Lanka				Nepal				Bangladesh			
	National Level				Maharashtra				Andhra Pradesh				Federal Level				Sindh				Punjab				National Level				National Level				National Level			
	In	Out	Bet	Cl	In	Out	Bet	Cl	In	Out	Bet	Cl	In	Out	Bet	Cl	In	Out	Bet	Cl	In	Out	Bet	Cl	In	Out	Bet	Cl	In	Out	Bet	Cl	In	Out	Bet	Cl
(a) Funding network																																				
UN & Multilaterals																																				
*UNICEF*	*X*	*5*	*6*	*3*	*X*	*4*	*7*	*10*	*X*	*4*	*6*	*7*	*X*	*1*	*2*	*1*	*X*	*2*	*4*	*5*	*X*	*4*	*3*	*6*	*X*	*2*	*2*	*4*	*4*	*1*	*2*	*2*	*X*	*3*	*2*	*6*
UNDP					X	X	X	X	X	X	X	X																								
World Bank	X	X	X	10	X	5	9	8	X	8	8	X					X	3	7	7	X	X	X	X	X	4	X	X	X	4	3	7	4	7	7	4
WFP																									X	5	X	X	X	3	4	5				
*WHO*	*X*	*X*	*X*	*9*	*X*	*X*	*X*	*X*	*X*	*X*	*X*	*8*	*X*	*2*	*3*	*2*	*7*	*1*	*1*	*1*	*10*	*2*	*2*	*5*	*X*	*3*	*5*	*5*	*X*	*7*	*7*	*10*	*X*	*5*	*X*	*X*
Bilateral donors																																				
AusAID/DFAT													X	5	X	10					X	X	X	X												
DFID	X	X	X	X																	X	X	X	X					X	6	X	4	X	X	X	X
USAID	X	7	7	7	X	X	6	X	X	10	9	X					X	4	X	X	X	5	4	8					X	2	5	3	X	1	1	2
International NGOs																																				
ACF																													X	10	X	X				
HKI																													5	9	X	8	9	X	X	X
MI																					2		7	7									X	X	X	X
Save the Children	X	10	X	X																	X	8	8	9					X	8	8	9	X	X	X	X
World Vision																									X	X	X	X								
(b) Technical support network																																				
UN & Multilaterals																																				
*UNICEF*	*6*	*5*	*8*	*8*	*X*	*2*	*6*	*9*	*X*	*1*	*4*	*3*	*X*	*3*	*5*	*7*	*1*	*1*	*1*	*1*	*5*	*6*	*5*	*3*	*6*	*6*	*9*	*8*	*X*	*2*	*2*	*2*	*X*	*3*	*4*	*2*
UNDP					X	X	X	X																												
World Bank	X	X	X	X	X	X	X	X	X	7	X	X	X	4	6	8	X	X	X	X	6	7	X	5	10	5	5	5	X	9	X	10	X	10	X	X
WFP	X	X	X	X													X	8	X	8	X	X	X	X	X	X	X	9	X	5	3	4	X	9	X	X
*WHO*	*X*	*X*	*X*	*X*	*X*	*X*	*X*	*X*	*X*	*7*	*X*	*X*	*X*	*4*	*6*	*8*	*X*	*X*	*X*	*X*	*6*	*7*	*X*	*5*	*10*	*5*	*5*	*5*	*X*	*9*	*X*	*10*	*X*	*10*	*X*	*X*
Bilateral donors																																				
AusAID/DFAT																													X	X	X	X				
DFID																					X	3	6	7									X	X	X	X
USAID	X	X	X	X					X	X	X	X	X	6	X	10	X	X	X	X	X	10	X	X					X	6	10	9	X	5	8	9
*International NGOs*																																				
ACF																	X	X	X	9									X	X	X	X				
HKI																													4	X	X	X	X	X	X	X
MI	X	X	X	X	X	X	X	X					X	7	X	X					X	X	X	10					X	X	9	X				
Save the Children	X	X	X	X									X	5	X	9													X	4	4	5	X	X	X	X
World Vision																	X	X	X	X					X	X	X	X	X	X	X	X	X	X	X	X

In: In-degree centrality; Out: Out-degree centrality; Bet: Betweenness centrality; and Cl: Closeness centrality

If an organisation has not been identified as an important contributor in the Net-Map interviews for IYCN then it is marked with a ‘blank’ for that study site. If an organisation has been identified as an important contributor but not one of the 10 top contributors in respect to the corresponding network measure then it is marked with “X”. If an organisation has been identified as an important contributor and one of the 10 top contributors in respect to the corresponding network measure then it is marked with its chronological position among the 10 top contributors

*ACF* Action against Hunger, *AusAID* Australian Agency for International Development, *DFAT* Department of Foreign Aids and Trade, *DFID* Department for International Development (United Kingdom), *HKI* Helen Keller International, *MI* Micronutrient International, *UNDP* United Nations Development Programme, *UNICEF* United Nations International Children’s Emergency Fund, *WFP* World Food Programme, *WHO* World Health Organization

From the Net-Map analysis in Table 3, it is evident that UNICEF and WHO (highlighted with italic font) were the most influential contributors across all study sites in both the funding and the technical support networks. The United States Agency for International Development (USAID) and the World Bank were the second most influential contributors for both funding and technical support activities of IYCN programmes across all study sites. As seen in Table 3(a), the Action against Hunger (ACF) and World Vision were found only once as an important contributor for funding activities (Nepal and Sri Lanka sites, respectively). Table 3(b) reveals that AusAID/DFAT and UNDP were found only once as an important contributor for technical support activities (Nepal and Maharashtra (India) sites, respectively). Interestingly, the World Bank was identified as one of the most important actors in most sites, except for Pakistan at a national level for funding activities, and Pakistan at a national level and Andhra Pradesh (India), for technical activities.

Most of the organisations in Table 3(a) and (b) were marked with “X” for the in-degree centrality measure. This indicates that although they were an important contributor in the majority of the study sites they were not one of the 10 top contributors for in-degree centrality. Since in-degree centrality is a measure of receptivity, an “X” for these organisations reveals that they exert influence on other stakeholders (through technical support and funding support) rather than being influenced by them. For all study sites, UNICEF was one of the 10 top contributors to out-degree centrality, betweenness centrality and closeness centrality for both funding and technical support activities. Out-degree centrality indicates the activity of an actor in a network, betweenness centrality denotes an actors’ control for information flow, and closeness centrality of an actor reveals the reachability of that actor by other network actors. Thus UNICEF has had a high-level activity, control of information flow and reachability compared to other organisations for both funding and technical support activities. Through its various programs in more than 90 countries across the world, including the five South Asian countries of this study, UNICEF has been working to make good nutrition a reality for the children, families and communities that need it most [19]. WHO and the World Bank were, at many times, one of the 10 top contributors to out-degree, betweenness and closeness centrality in both IYCN funding and technical support networks. In 2008, the World Bank conceived the South Asia Food and Nutrition Security Initiative (SAFANSI) with support from numerous donors. Through this initiative it has provided platforms for enabling improvement in nutrition especially IYCF which has led to implementation of various interventions throughout the region that has in turn affected IYCF policy making in the region [20].

### Significance

This is the first attempt to systematically map the stakeholders across South Asia for IYCN and conduct a network analysis using rigorous research methodologies. The findings of this study are appearing at a critical time when there has been an increased interest and commitment to address the problem of undernutrition in the region. In all these countries, many governments as well as non-government actors have been involved in nutrition, including IYCN both for policy and programming. Understanding stakeholders and their relations in policy process and program related decisions will be useful for guiding the process for effective decision making and eventually for achieving the desired outcomes.

### Strength and limitation

A key strength of our study is that we collected network data to map stakeholders for IYCN from five South Asian countries, and both at the local and central levels for two countries. We have used appropriate methods (i.e. group interviews and Net-Map approach) for data collection, and for social network analysis (i.e. centrality measures). The limitations of our study include the subjective nature of the evaluation of the networks by the participants, albeit the mapping of stakeholders was done based on group interviews. In addition, the views of the participants might be influenced by their own experience and understanding of the policy and programmatic decision-making process related to IYCN in their countries. Another limitation of this study is that the interviews focussed more on mapping the stakeholders and their networking and it has not been able to capture the entire dynamics of relations in detail due to time constraints during interviews. This eventually gives an opportunity to conduct further in-depth studies to explore the relational dynamics of each of the actors within a range of stakeholders in the network and how they can be linked with shaping policy and programmatic decisions for IYCN in each country.

## Conclusion

This study followed a social network analysis approach for analysing two different types of stakeholder networks (i.e. funding and technical support networks) in nine different study sites across five different South Asian countries. It identified major international stakeholders in terms of level of activity, reachability by others and capacity to control the flow of information within the network. Government stakeholders were more engaged in technical support activities, they received more support for funding and technical support activities from other stakeholders compared with the support that they offered and there was no significant difference in their level and pattern of engagements across different study sites. Understanding what organisations are contributing to funding and technical support allows governments to plan how to develop their own institutional capacity to shape IYCN policies in the future.
